# Dynamic near-term risk prediction of clinically significant immune-related adverse events using longitudinal electronic health records

**DOI:** 10.64898/2026.10.06.26364907

**Published:** 2026-10-08

**Authors:** Liyuan Gong, Alice Agyekum, Nitish Aswani, Harry Reyes Nieva, Chin Hur

## Abstract

**Objective:**

To develop and evaluate a dynamic model that repeatedly estimates the 30-day risk of a first grade ≥2 immune-related adverse event (irAE) during immune checkpoint inhibitor (ICI) therapy.

**Materials and Methods:**

Adults initiating ICI therapy during 2015-2022 comprised the development cohort; 2023 initiators formed a nonoverlapping internal temporal evaluation cohort. Every 21 days during the first treatment year, models used available EHR information to predict a first structured phenotype-defined grade ≥2 irAE during the next 30 days. Evaluation included pooled and within-landmark discrimination, precision–recall performance, calibration, and patient-clustered bootstrap confidence intervals. Feature-state ablations and alternative update schedules examined the contribution of dynamic information.

**Results:**

Development included 1,522 patients and 18,744 evaluable landmarks; temporal evaluation included 526 patients and 6,263 landmarks, of which 184 were positive (2.94%). The multiscale XGBoost model achieved pooled AUROC 0.716 (95% CI, 0.676-0.755), AUPRC 0.067, within-landmark AUROC 0.672 (95% CI, 0.631-0.716), Brier score 0.0281, and log loss 0.1245. The time-aware baseline achieved AUROC 0.631 and within-landmark AUROC 0.536. Updated treatment exposure and laboratory trajectories accounted for most of the gain. A complete-state extension achieved AUROC 0.719 and AUPRC 0.073. The 21-day schedule had the highest discrimination among tested cadences.

**Conclusion:**

Longitudinal EHR updating improved near-term irAE prediction beyond pretreatment characteristics and treatment time. Phenotype adjudication and independent evaluation of calibration, transportability, and clinical utility are needed before implementation.

## Full Text Availability

The license terms selected by the author(s) for this preprint version do not permit archiving in PMC. The full text is available from the preprint server.

